# Characteristic processes of human evolution caused the Anthropocene and may obstruct its global solutions

**DOI:** 10.1098/rstb.2022.0259

**Published:** 2024-01-01

**Authors:** Timothy M. Waring, Zachary T. Wood, Eörs Szathmáry

**Affiliations:** ^1^ Mitchell Center for Sustainability Solutions, University of Maine, Orono, ME 04469, USA; ^2^ Department of Biology, Colby College, 4000 Mayflower Hill Drive, Waterville, ME 04901, USA; ^3^ Institute of Evolution, Centre for Ecological Research, Budapest, Hungary; ^4^ Center for the Conceptual Foundations of Science, Parmenides Foundation, Pöcking, Germany; ^5^ Plant Systematics, Ecology and Theoretical Biology, Eötvös University, Budapest, Hungary

**Keywords:** ETII, Anthropocene, evolutionary transition, cultural evolution, sustainability, human evolution

## Abstract

We propose that the global environmental crises of the Anthropocene are the outcome of a ratcheting process in long-term human evolution which has favoured groups of increased size and greater environmental exploitation. To explore this hypothesis, we review the changes in the human ecological niche. Evidence indicates the growth of the human niche has been facilitated by group-level cultural traits for environmental control. Following this logic, sustaining the biosphere under intense human use will probably require global cultural traits, including legal and technical systems. We investigate the conditions for the evolution of global cultural traits. We estimate that our species does not exhibit adequate population structure to evolve these traits. Our analysis suggests that characteristic patterns of human group-level cultural evolution created the Anthropocene and will work against global collective solutions to the environmental challenges it poses. We illustrate the implications of this theory with alternative evolutionary paths for humanity. We conclude that our species must alter longstanding patterns of cultural evolution to avoid environmental disaster and escalating between-group competition. We propose an applied research and policy programme with the goal of avoiding these outcomes.

This article is part of the theme issue ‘Evolution and sustainability: gathering the strands for an Anthropocene synthesis’.

## 1. Introduction

Our species has come to dominate Earth's ecosystems. This state has been termed the ‘Anthropocene’ [1], a geological epoch defined by stratigraphic signatures of human activity such as concentrations of carbon dioxide and radioisotopes [2]. In this period, humans have changed global ecological processes [3] and shifted in the interactions between ecosystem processes and evolution in countless species [4].

The Anthropocene creates a novel evolutionary condition for both the biosphere and our species. Our exponential population growth, dramatic environmental modification and technological systems have created a novel evolutionary environment for humanity, which may entail existential risk for humanity [5]. Also, human environmental impacts are on course to constitute one of the larger extinction events over approximately 3.7 billion years of life [6]. However, while mass extinctions are mostly thought to be caused by violent non-biological causes, such as volcanism or impact events, the current mass extinction (i.e. [7]) appears to be a biogenic event caused by a single species.

Research on human environmental impacts has mostly overlooked the role of human evolution. Likewise, contemporary visions for environmental stewardship (e.g. [8]) are rarely informed by either human evolutionary history or current evolutionary mechanisms. When evolutionary theory is invoked, it is often used as a metaphorical tool rather than a useful mechanistic theory of change (e.g. [9,10]). Currently, global environmental research does not commit to an evolutionary understanding of human behaviour or integrate the evolutionary history and processes that have resulted in the global-scale impacts of human societies. Like Ehrlich & Ornstein [11], we propose that understanding human evolution is key for understanding the causes and progression of the Anthropocene as well as for the effort to design a livable future.

Research that does connect human evolution to anthropogenic environmental impacts highlights two key factors: the role of culture and the importance of group structure and cooperation [12,13]. The evolution of human culture (including language) is widely understood to be a central feature of human evolution (e.g. [14]) and is increasingly recognized as important in understanding human environmental behaviour [15]. However, the role of culture in human environmental impact is complex. On one hand, as Ehrlich & Ehrlich [16, p. 781] suggest: ‘Humanity created the Anthropocene through cultural evolution’. On the other, human culture is seen as necessary to achieve sustainability through cooperative solutions [10,17,18]. Thus, adaptive cultural evolution is implicated both as a cause of global environmental decline, and the key to spread policies and solutions to mitigate anthropogenic impacts [19].

The evolution of group-level cultural traits via cultural group selection [20,21] is a central candidate mechanism to explain the evolution of human environmental exploitation [22]. Ellis proposes that the cumulative feedbacks between cultural niche construction and cultural group selection have led to the human domination of the biosphere by selecting for groups that exploit natural resources ever more efficiently and at ever greater scales [3,23]. Theoretical models have shown that cultural group selection can generate sustainable resource use behaviour in simple systems of groups under conditions of territorial resource control [24,25] and empirical research has begun to bear this out [26,27]. However, human prosociality is expansive and goes beyond simple parochial patterns of cooperation. For example, humans readily form cooperative connections and exchange cultural elements between groups [28]. Between-group cooperation can take the form of trade networks, military alliances and treaties, and may often be coupled with cultural transmission including the sharing of language and traditions. These long-distance between-group interactions may play a role in natural resource management and environmental exploitation as well [29]. Human capacity to grow new cooperative and cultural connections between groups may even result in the formation of new social units at a larger scale. Therefore, based on what we know of the evolution of human culture, cooperation and groups, it remains unclear what the prospects for global cooperation in environmental management are. This paper is a contribution to a more complete theory of how human evolution gave rise to and may unfold during the Anthropocene.

It has been proposed that human evolution can be described as an evolutionary transition in inheritance and individuality (ETII) [30]. In this paper, we develop the hypothesis that the human domination of the biosphere is a unique consequence of this ongoing human evolutionary transition and explore the implications. Our effort is in the spirit of developing the novel theory necessary for the unprecedented challenges of our time [31].

## 2. Human evolutionary ratchets help explain the Anthropocene

Many agree that human evolution may be partly defined by some kind of evolutionary transition [32–36]. These proposals differ on whether the transition hinges on individuality or inheritance. For example, while protolanguage may have appeared in *Homo erectus* and catalysed human evolution [37], the ‘social protocell’ model [36] depends on differential reproduction of cultural groups with heritable institutions [38]. For Powers *et al*. [35], the emergence of culturally determined institutions marks the central transition. Others posit that a transition in individuality is ongoing and may culminate in the future [34] or even involve an egalitarian transition joining humans with artificial intelligence [39]. The ETII hypothesis builds on these proposals. It posits that long-term human evolution is driven by a shift in the primary mechanism of evolutionary inheritance from genes to culture [30], caused by the greater adaptive power of cultural evolution in humans (e.g. [14,40–42]).

**Figure 1.**
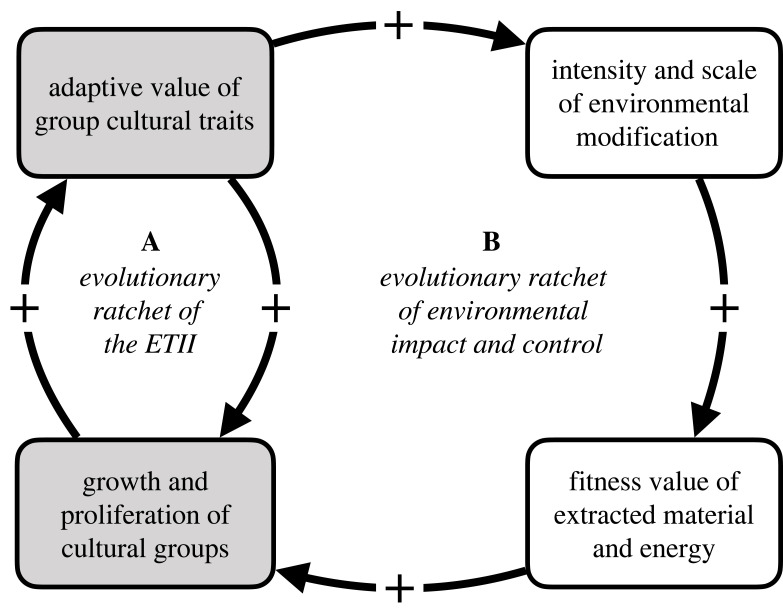
An evolutionary ratchet of environmental intensity. The positive feedback system of the ETII (A) entails additional positive feedback (B), in which group-level cultural innovations in environmental management tend to increase the scale and intensity of environmental modification and extraction, which in turn accelerates the proliferation of cultural groups with those innovations.

The hypothesis suggests that human evolution is dominated by a positive feedback between the adaptive capacity of human culture, which generates group-level adaptations, and the strength of human groups which employ them (figure 1*a*). This evolutionary ratchet generates two central patterns. The first is a shift in the bulk of adaptive information from genes to culture. This pattern, called ‘*fitness export*’ [43,44], occurs as the human reliance on group-level social and technological adaptations increases, causing selection on human genes to wane. The second is a shift in human organization from individuals to groups, a defining pattern of evolutionary transitions in individuality (ETIs) generally [45]. This shift is characterized by increases in the spatial and social scale of societies and their degree of internal cooperation. Like other ETIs, the human ETII involves a positive feedback mechanism [46] which drives the emergence of a higher level of organization and individuality. Unlike other ETIs, a hypothetical ETII would conclude with the emergence of a population of *cultural superorganisms* and is therefore novel in many regards. The ETII hypothesis is supported by evidence for group-structure and cultural adaptation in human evolution [20,47–49], but raises questions about the evolution of human interaction with the environment.

We have argued that the evolutionary ratchet of the ETII helps to explain patterns of human evolution past and present [30]. Cumulative cultural evolution is believed to have strong evolutionary ratcheting effects [50,51]. Here, we explore how the same ratcheting feedbacks might additionally generate interactions between human evolution and ecology in the past, present and future. The ecological setting is crucial for the understanding of particular major transitions in general [52].

The logic connecting human domination of the biosphere to the ETII is simple. The evolution of human societies has been typified by positive feedback between the adaptive capacity of human groups and their growth and proliferation (ETII ratchet, figure 1,A). These dynamics entail positive feedback between the scale and intensity of environmental resource use and the adaptive benefits human groups extract from those resources (environmental ratchet, figure 1,B).

Proposals for the Anthropocene geological epoch have placed its onset in the mid-twentieth century [2] with the global sedimentation of novel inorganic residues. However, these recent global-scale impacts are clearly part of a larger trend in human environmental impacts and the scale of human societies with roots millions of years in the past. This trend, we propose, is the ETII.

This cumulative process extends from the emergence of cultural transmission in the *Homo* lineage and includes collective environmental practices such as cooperative hunting [53], herding [54], fishing and agriculture [55]. Ongoing iterations of growth, elaboration and expansion have resulted in both continental-scale societies of immense power and the global environmental impacts they have engendered.

## 3. The evolution of the human ecological niche

The relationship between organisms and their environment is typically described through the concept of the *ecological niche*. In classical niche theory, the niche is the set of abiotic and biotic conditions under which a species can survive and reproduce at a population-sustaining rate [56]. Organisms can also modify their environment to make it more favourable for themselves—this process is known as *niche construction* [57]. Humans are perhaps the ultimate niche constructors in terms of our environmental modifications. In humans, niche construction is largely the result of accumulated cultural adaptations for environmental modification and resource extraction. This has been termed *cultural niche construction* [58–60]. Over evolutionary history, the human niche has changed dramatically (table 1), growing from that of a primate omnivore to a planetary-scale niche constructor [72], affecting nearly every aspect of ecology [73], evolution [74] and eco-evolutionary dynamics [4] in the natural world.

**Table 1.** Changes in the ecological niche of human groups over evolution. The ETII hypothesis puts the human domination of the biosphere into a long-term evolutionary perspective, with the emergence of systems of environmental modification and control of increasing intensity and scale. Population estimates from Morris [61] and Klein Goldewijk *et al*. [62]. For additional detail, see table 3 in Ellis [3].

period	pre-transition	I	II	III	IV	V	VI		post-transition
state	most recent common human ancestor	hominin expansion	modern human expansion	global expansion	agricultural revolution	industrial revolution	the Anthropocene	…	hypothetical cultural superorganism
realized ecological niche	primate omnivore	primate omnivore and scavenger, hunter	apex omnivore	apex omnivore, ecosystem engineer	human-dominated landscapes with domesticates, organic metabolism	human-controlled landscapes with organic/inorganic metabolism	human-controlled landscapes with organic/inorganic metabolism	**…**	(unknown)
start of period	approximately 3 Ma [63,64]	>2 Ma–0.5 Ma	approximately 200 kya [65]	approximately 70 kya	approximately 10 kya	approximately 220 ya	present	**…**	(unknown)
species population	NA	NA	NA	approximately 2 000 000	approximately 4 400 000	approximately 990 000 000	approximately 8 000 000 000	**…**	(unknown)
population of largest city	NA	NA	NA	NA	approximately 1000	approximately 1 000 000	approximately 25 000 000	**…**	(unknown)
spatial scale of largest society	NA	NA	NA	local camps, <1 km^2^ [66]	sub-regional	regional	continental	**…**	(unknown)
environmental impact intensity	equivalent to other primate species	greater than other primate species	much greater than other primate species	global expansion, megafaunal extinctions	regional driver of ecosystem function, species evolution, artificial selection	regional driver of ecosystem function, species evolution, inorganic pollution, mass extinction	global driver of ecosystem function, species evolution, gene modification, inorganic pollution, mass extinction	**…**	(unknown)
group-level traits of environmental control	NA	local collective hunting and scavenging [67–69]	local cooperative hunting [70], fishing and gathering, fire control [71]	territorial cooperative food provision, shelter and defence	sub-regional domestication, cities, organic fertilizer, harvesting restrictions, stone architecture, canal irrigation	regional eradication policies, national environmental law, nature preserves	continental national environmental fines and taxes, genetic modification, anti-extinction policies, global environmental law	**…**	(unknown)

As cultural adaptations for niche construction have accumulated over evolutionary history, the scale and intensity of human environmental impacts have grown in tandem. Prior to human cultural and linguistic abilities, human ecological impacts were not substantially different from that of other large primates. That changed when hominin species developed collective scavenging and hunting behaviours somewhat prior to 2 Ma [67–69]. A strategy of confrontational scavenging may even have predated *H. erectus* [64]. These collective strategies were probably facilitated by protolanguage and vice versa [75,76]. The global expansion of modern humans beginning approximately 200 kya [65] represented another change in the human niche. Human expansion was probably caused by improvements in culturally coordinated group behaviour and the cultural transmission of fire making methods and resulted in the extinction of numerous large mammals [77,78]. These cultural and group-level characteristics enabled a transition to cooked food with greater nutrient availability, and fewer toxins and pathogens. Increases in carnivory probably helped early humans expand their geographical niche [79]. Niche construction continued with the domestication of dogs (*ca* 23 kya, [80]) and cattle (*ca* 11 kya, [81]). Human impacts grew significantly with the advent of agriculture at the start of the Holocene *ca* 11.5 kya [82,83]. The emergence of agricultural societies with heavy local impacts including irrigated farmland, controlled pastures and cities contributed to a global transformation of land use by 3000 ya [84]. The industrial revolution marked the emergence of group-level inorganic metabolism in which human groups vastly expanded their ability to control inorganic energy and materials, giving societies more power, and greater control over the environment. Industrial technology resulted in new types of impacts including chemical pollution, ozone degradation, fishery collapse, landscape modification, groundwater depletion, anthropogenic drought, toxic pollution, radioactive waste, anthropogenic climate change and others.

A few observations can be made. First, the size of human groups have increased by eight orders of magnitude over human evolution. Second, the scale and intensity of environmental resource use and concomitant impacts have also grown dramatically over human evolution, reaching and exceeding global limits in some cases (i.e. [85]). The dramatic growth in the scale of the human ecological niche implies that evolutionary interactions between human groups have also changed. Evolutionary competition between human groups was initially low as groups were small and sparse. Most of human evolution can therefore be characterized as an eco-evolutionary regime of *indirect resource competition* between human groups in which no group could influence the global environment or all other groups. While inter-group competition and parochial altruism are thought to have played an important role in the evolution of hunter–gatherers (e.g. [86]), societal interactions (including trade and warfare) and environmental modifications remained local or regional. After the agricultural revolution, inter-group war became larger, more structured and more important in human evolution [87]. Following the industrial revolution, societal interactions including communication, trade, war, diseases and environmental interactions became increasingly global. Also, the survival and status of human groups became globally interdependent [88,89]. This began a new eco-evolutionary regime of *direct resource competition* in which single groups can strongly influence the global environment, and potentially all other groups. This novel eco-evolutionary condition for human groups typifies the Anthropocene. Three salient examples of this interdependence are anthropogenic climate change, the COVID-19 pandemic and the proliferation of society-ending nuclear weapons. Also, while the scale and impact of human groups has increased, the finite resources of Earth have not, which suggests that human groups may not yet be well adapted to the novel conditions of the Anthropocene.

## 4. Group-level cultural traits for environmental control

A general trend also emerges from table 1. *Group-level cultural traits* for direct environmental control have emerged in parallel with each new expansion in the environmental scale and intensity of human societies. These include *extractive traits* such as irrigation systems, forest harvesting machines and mechanized agricultural technology as well as *management traits* such as water quotas, forest use regulations and pesticide laws. Early human traits for environmental control are exemplified by coordinated food collection such as cooperative hunting [67–70], fishing and gathering that emerged in the Pleistocene. The emergence of agriculture in the Holocene involved more complex group-level environmental control traits including canal irrigation [90], harvesting restrictions, stone architecture, transportation and cities. Later, the industrial revolution was accompanied by even more complex traits, including continental trade and transport networks, eradication policies for nuisance species and diseases [91], agricultural subsidies, national environmental laws, natural space protections, environmental regulation, pollution fines, genetic modification, anti-extinction policies and the emergence of global environmental law [92].

Group-level environmental management traits are also evolutionarily novel, and the nature of and constraints on the evolution of these traits are poorly understood. Following our conceptual model in figure 1, novel technologies and systems for resource extraction (e.g. whaling ships) could enable the expansion of human groups. Larger groups encounter new challenges in managing the environment at the larger scale (e.g. decline of whale populations) and new opportunities (e.g. fossil fuel energy). Over time, groups may learn and evolve new traits, technologies and systems for controlling and sustaining these resources (e.g. whaling limits treaties), as well as new extractive technologies (e.g. fossil fuel combustion technology). In this hypothetical process, extractive traits emerge first, followed by societal growth. This may sometimes lead to constraints on extraction driven by an ongoing need energy and materials. So, the scale of *management traits* should often lag behind the scale of societal organization. For example, continental air quality laws can only emerge after a society has reached the continental scale.

Increasingly, policy scholars suspect that in the case of global resources such as atmospheric carbon dioxide or ocean pollution, sustainable management will require a complex set of *global cultural traits for environmental management*, including novel social, technical, and legal systems at the global scale (e.g. [93,94]). Thus, it would seem that planetary sustainability could most-readily be achieved by a global-scale cultural group with the proper group-level cultural traits. This is why Corning [95] suggests that a global superorganism may be necessary to tackle global climate change.

One early theory of global environmental sustainability is the Gaia hypothesis [96]. It suggests that Earth systems and the biosphere have evolved to become collectively self-regulating. But evidence does not support the original Gaia hypothesis [97]. In evolution, complex evolved traits necessary to maintain homeostasis emerge from long-term adaptive evolution among a population of self-organized systems rather than from self-organization alone. New versions of Gaia theory have argued that self-regulating ecological features could emerge via sequential selection for ecological persistence if communities can flexibly reassemble in a resilient biosphere [98–100]. However, global-scale reorganization would be very slow and persistence selection would probably oppose that of selection on sub-global groups. Therefore, the persistence selection mechanism would have to be strong enough to override selection on the rapidly evolving sub-global human groups, which is highly unlikely. Thus, even the newer Gaia theory does not provide a plausible route to global sustainability. Moreover, neither theory incorporates human cultural evolution.

## 5. The population structure problem of the human evolutionary transition

We can use this basic understanding of the evolution of group-level environmental management to explore the prospects for a sustainable or well-managed biosphere. As highlighted above, the hypothetical ETII engages an evolutionary ratchet that may have led human culture towards increasingly profound environmental impacts, at increasing spatial scale with consequences for the health of the biosphere and even the long-term survival of our species. Here, we argue that our specific evolutionary path will determine the fate of human–environment interactions and global sustainability. Our concern derives from four observations:

(i) *human evolution is driven by group-level cultural evolution in the very long term*: the ETII suggests that human evolution is driven by group-level cultural evolution [30], which requires a population of groups [21,20]. Evolutionary competition between groups is a key process in ETIs that strengthens group-level identities and solidifies group-level control of individuals and resources [45];
(ii) *global sustainability requires global cultural traits for environmental management:* the sustainable regulation of a planetary biosphere would appear to require a refined and complex set of technical and legal systems and behaviours (e.g. [94]), and would include and enforce cooperation between groups. These global cultural traits could be expressed by a single global society or global-scale individual. Such global cultural traits could evolve through adaptive cultural evolution among a population of global-scale entities;
(iii) *the scale of environmental management traits lags behind the scale of society:* extractive traits may precede the expansion of human groups. However, cultural traits for environmental management at a given scale seem to emerge most robustly within societies of that scale and therefore only evolve after those societies form. For example, strong fines for pollution are more common within nations that between them; and
(iv) *evolution in a population of sub-global groups favours the emergence of sub-global traits for environmental control*: Earth supports a population of evolving sub-global groups. Evolution among entities in a shared environment favours adaptations for resource competition and extraction (e.g. [101]). Thus, there can be limited collective action towards environmental stability in the face of evolutionary competition between groups.

Therefore, the population structure necessary for the adaptive evolution of global cultural traits for environmental regulation is in conflict with the current and historical evolutionary processes acting on human groups. Phrased another way, competition among cultural groups precludes the evolution of global systems to sustainably manage the planet. Cultural group selection could plausibly generate adaptive global cultural traits if operating among groups with sovereign control over separate planets. However, this is not likely to occur in our species in the foreseeable future, particularly before the worst effects of our global impacts (such as climate change) are felt. This idea is supported by mathematical models which show that environmental patchiness is often necessary for successful evolutionary transitions [102]. The problem is we have only one patch.

This is the *population structure problem of the human ETII*. To evolve the traits necessary to maintain a sustainable planetary environment, a population of global-scale societies is required. Short of gaining access to new livable planets, this problem appears to have no simple solution and poses mounting dangers for human survival and biosphere stability in the coming millennia.

## 6. Navigating human evolution in the Anthropocene

The Anthropocene and the ETII are linked by the scale of environmental control and cooperation they imply or require. The ETII operates on human cooperation over time, which global environmental challenges require. Also, the Anthropocene is defined by its global scale, a scale that human impacts have recently breached. Thus, we reason that factors and changes that influence human evolution and the ETII will have relevance for global environmental challenges, and vice versa.

**Figure 2.**
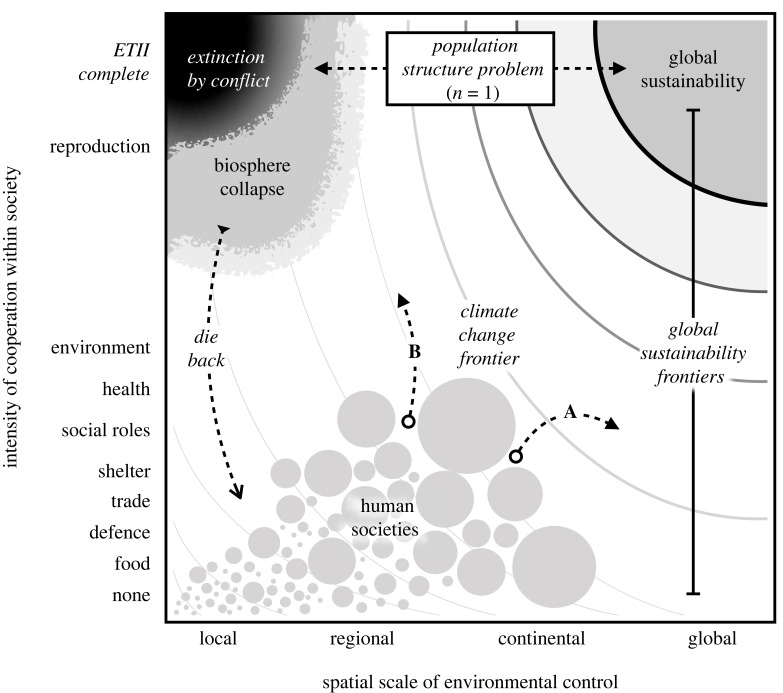
Dimensions of environmental management create an attractor landscape for long-term human evolution. Environmental sustainability challenges (curved frontiers) require a minimum level of cooperation in a society of a certain minimum spatial size. Alternative potential paths move humanity toward different long-term evolutionary outcomes. In path B, competition between societies over common environmental resources creates cultural selection between groups for increasingly direct competition and conflict. Path A, growing cooperation between societies facilitates the emergence of global cultural traits to preserve shared environmental benefits.

Any domain of environmental management can be characterized according to two variables: the spatial scale over which the environmental resource must be sustained, and the level of cooperation necessary to benefit from the resource in a durable manner. Sustainability challenges require a minimum level of cooperation in a society of a certain minimum spatial size. For example, to solve local lake pollution, the level of cooperation needed is only that which is sufficient to stop pollution among the lakeshore residents, while to solve groundwater management, cooperation is needed among groundwater users of a watershed [103]. If these users are farmers who depend on groundwater irrigation for their livelihoods, the required level of cooperation is very high as cooperation might entail major economic loss. In this way, each environmental resource can be visualized as a *sustainability frontier* (figure 2).

Each new frontier presents an adaptive challenge that requires the development of novel social and technological arrangements (i.e. cultural traits) for environmental control at new and greater scales. Human societies have experienced many sustainability frontiers in the past, solving some (e.g. maintaining captive populations of food species, supplying nutrients for crop growth) and failing others (e.g. watershed pollution, biodiversity protection). The Anthropocene is characterized by global sustainability frontiers (disease, water, climate change, antibiotic resistance, zoonotic diseases and pandemics) which remain to be solved.

Each axis in figure 2 has a characteristic societal dynamic. The *y*-axis represents intensity of cooperation within groups. A general finding from the evolution of cooperation [45] and cultural multi-level selection in humans [20,47] is that cooperation in a group is often driven by competition between groups. Therefore, *increases in between-society competition tend to move societies along the y-axis* (i.e. [36]). For example, fierce competition between gangs of lobstermen seems to have driven the evolution of within-gang cooperation via cultural group selection [26]. The *x*-axis represents the spatial scale of resources and human groups. The spatial scale of a resource determines the spatial scale of a society capable of sustainably using it. For example, the Roman empire was among the first to build large-scale aqueduct infrastructure, because it occupied the territory necessary to control large-scale water distribution in part through the growth of political hierarchy. Therefore, *increases in hierarchical organization tend to move societies along the x-axis*.

Over human evolution, societies have grown in scale of environmental control, intensity of cooperation and in population size (figure 2, lower left). The evolutionary ratchet of the ETII hypothesis suggests that human evolution is unlikely to remain in this quadrant.

Hypothetically, a completed ETII would produce cultural superorganisms: societies with total and complete cooperation, including, presumably, the group-level reproductive centralization observed in eusocial insects. If an ETII were to complete, the transition might unfold over thousands to millions of years across this planet or many. However, the ETII may fail. Its core feedback mechanism of evolutionary competition between human groups in a shared environment could drive humanity to extinction through multiple scenarios (figure 2, top, population structure problem). By comparison, success in climate change requires a much lower level of cooperation (agreement to use certain energy sources) and could unfold relatively rapidly via cooperation between societies. However, solving global climate change does require social coordination at a global scale (figure 2, right).

Using figure 2, we can evaluate potential evolutionary paths for their sustainability outcomes, existential risks and likelihoods. We focus on two contrasting paths characterized by different eco-evolutionary processes. We consider these paths equivalent to the dynamic adaptive policy pathways approach of [104]. Future evolutionary paths are unknown and inherently stochastic and reversible. Societal failures from environmental damage have occurred [105–107]. Countless paths are possible. Societies may grow and proliferate, shrink and die off. We highlight two evolutionary paths (path A and path B) which represent alternative possible futures of interest. Both paths start from our species current position and move in different directions relative to global sustainability frontiers, and to the long-term outcomes of human evolution.

### (a) Path A

Growing cooperation between societies facilitates the emergence of global cultural systems of environmental control necessary to solve shared challenges such as climate change. This is the sustainable and desirable path. It relies on the bottom-up self-organization of systems of global environmental governance, and voluntary expansion of cooperation between groups and societies. Although prior societal expansion may have occurred in this mode, it is not congruent with a human ETII. Specifically, path A is not favoured by evolutionary processes for two reasons. First, selection on groups operates against cooperation between groups. Second, the population structure problem described above suggests that accumulating adaptive variation in global cultural traits is unlikely.

### (b) Path B

Growing competition between societies over environmental resources accelerates the evolution of traits for direct competition and conflict. This undesirable path has significant evolutionary momentum. As we have detailed, much of recent human evolution has been characterized by between-group competition driving the growth of within-group cooperation and hierarchy [48,87,108,109]. However, path B is distinguished from prior evolutionary history because it occurs in a state in which direct environmental competition becomes increasingly unavoidable. In the short term, path B could result in major ecological collapse and human dieback as groups become more powerful but not more integrated (figure 2, left).

More problematic is that path B creates a self-reinforcing (positive) feedback system which selects for ever more competitive human groups. Positive feedbacks are probably a common feature in ETIs [46]. This competitive feedback could accelerate an ETII in humans at the sub-global scale. Feedback could progress from a mere lack of willingness to engage in between-group cooperation over global environmental regulation (indirect competition), into direct competition over environmental resources, and finally into survival competition and outright military conflict. Warfare selects for aggressive and expansionist group-level cultural traits and destructive technologies. For example, the emergence, refinement and proliferation of nuclear weapons were driven by conflict (World War II, The Cold War) between nations. If human evolution in an ETII becomes characterized by this type of evolutionary competition, it could lead to intense global warfare among increasingly aggressive groups, and even mutual destruction and human extinction in the very distant future. To clarify, our species may not be at immediate risk of extinction. Some humans might even survive a global nuclear winter. However, our social structure and way of living is probably in near-term danger.

However, how realistic is path B? We do not yet have sufficient evidence to evaluate this question. Recent meta-analysis supports multiple causal connections between resource interactions and war [110]. In their evaluation of the global catastrophic risks (GCRs) facing humanity, Fisher & Sandberg [111] counted 15 of 18 GCRs as anthropogenic—they believe humanity may face more categories of risk from its own actions than from any external source. Thus, path B could lead to biosphere collapse in the short term or extinction in the very long term (figure 2, upper left). As Søgaard Jørgensen *et al*. [112] argue, the Anthropocene may contain evolutionary traps for humanity. If so, path B is the largest and final trap. It should be our desperate goal to avoid such a path at all costs.

## 7. The expansive nature of human sociality

Human sociality is uniquely expansive, and so it may be that the historical upward trajectory in the scale and intensity of cooperation can continue into the Anthropocene, avoiding the worst outcomes we have described. Human groups often cooperate and share cultural elements even in the absence of external pressures (see [29]), creating a fitness interdependence which may mitigate competitive outcomes. Perhaps the expansive quality of human sociality may mitigate this scenario.

### (a) Between-group cultural transmission

The global transfer of cultural elements today is beyond that of any other era, with internet connectivity increasingly ubiquitous. Perhaps such between-group cultural transmission could facilitate the emergence of a global social identity which could support the development of necessary global sustainability traits (see [113]). However, between-group transmission may often reinforce cultural group selection [20], which is centrally implicated in the cumulative cultural evolution of extractive traits including fossil fuel technology. So, between-group cultural transmission provides no escape from the accelerating feedbacks of path B.

### (b) Trade

Trade is a strong type of between-group cooperation and an important force in human society and evolution. Human trade is akin to niche partitioning, in which ecological competition promotes the evolution of clearly separated niches, reducing future competition [114]. Humans have traded for possibly hundreds of millennia (e.g. [115]). However, trade is generally thought to emerge in positive-sum conditions when there are ‘gains to trade.’ Also, without effective regulation, trade can generate negative environmental and social externalities, particularly in industrial economies. Indeed, the success and growth of global trade appear to be a primary driver of the environmental crises of the Anthropocene. So, if trade is used as part of solutions, it must be applied with great care.

### (c) Collective environmental governance

Research the on emergence of collective environmental governance, exemplified by Ostrom [103], reveals that human culture sometimes evolves to modify the conditions of resource conflict to facilitate sharing, conservation and mutually beneficial outcomes. Emergent self-governance also occurs between groups, such as in formation of international treaties, providing some hope for global environmental governance. However, the critical precondition in models of the cultural evolution of sustainable environmental governance [24,25] is the availability of locally controllable resources, which give group fitness value to cultural traits for environmental management. On the other hand, such models do not include complex cognition or foresight, so humans may solve collective challenges more readily than current models predict. However, it may be, our argument is that if human society can create similar institutions at the global level, we will need to do so not only without the assistance of adaptive group-level cultural evolution, but in spite of it.

If human sociality emerged from an evolutionary transition in inheritance and individuality as has been proposed [30], then there is nothing in its expansive nature which could offer any escape from the evolutionary challenges we have described in the Anthropocene. It remains to be seen how well the expansive nature of human sociality can counterbalance reductions in the scope for mutually beneficial environmental cooperation.

## 8. Research agenda

**Figure 3.**
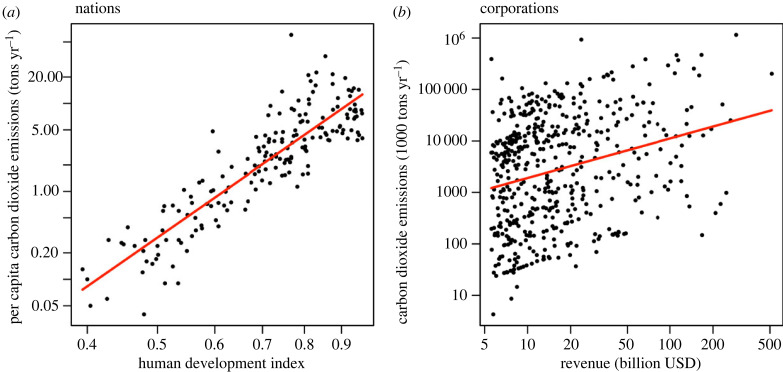
Environmental impact increases with proxy measures of group fitness. (*a*) Across nations, per capita carbon emissions (2021 data: [127]) are strongly correlated with the Human Development Index (2020 data: [128]). Other measures of wellbeing could also be used. (*b*) Across large corporations, total carbon emissions (2019 data: [129]) are correlated with corporate revenue (2015 data: [130]). Data do not include scope 3 emissions from the supply chain. These correlations are at least partly causal because fossil fuel consumption remains the easiest way to increase energy consumption and immediate quality of life. These relationships suggest that both types of human group are probably experiencing group-level cultural selection for increased carbon emissions. To solve anthropogenic climate change, the direction of selection needs to be reversed.

We propose a novel research agenda aimed at understanding the constraints on human evolution in a limited biosphere. Rigorous theoretical and empirical research on this topic might help humanity avoid potential catastrophes. We propose a series of research questions ranging from general and theoretical topics to pressing and applied matters:

(i) *does the human ETII hypothesis have internal validity?* Basic research on the ETII is necessary. A theoretical model of the human ETII is needed to test the internal validity of Waring & Wood's [30] theory. Such models could draw on existing models of human ETIs (e.g. [38,102,116]) and endogenous cultural group selection [24]. In addition, we might study the processes and constraints on previous ETIs in other systems. For example, it may be possible to draw useful analogies between the genetic kin selection in fraternal ETIs such as the evolution of multicellularity [102,117] and the evolution of eusocial termites approximately 150 Ma [118], and the ‘cultural kin selection’ expected in a human ETII;
(ii) *how strong is cultural group selection in humans?* To date, empirical evidence for adaptive group-level cultural evolution has been largely driven by case studies [20,26,119], and few quantitative studies have been performed (see [47]). However, large temporal datasets are increasingly easy to construct for recent history, and approaches such as the SESHAT global history databank methodology [120] can be used for deep historical datasets, and archeological research could be employed to estimate evolution among human groupings and settlement types over large time periods of the past;
(iii) *how strong is cultural group selection for sustainable environmental traits among nations and corporations?* Synder's [121] hypothesis that humans evolved culturally (and perhaps genetically) to be unsustainable can be tested empirically. Earth only supports a small population of nations (approx. 200), with a slow generation time (approx. 250 years, see [122–124], but it houses an estimated 300 million companies [125], with an average longevity of less than 20 years [126]). Thus, we expect the rate of adaptive cultural evolution by selection and cultural learning to be dramatically faster in companies than in territorial governments. In figure 3, we demonstrate how current group populations can be studied with carbon footprint data;
(iv) *how can cultural evolution among corporations and nations be harnessed to reduce global environmental risks?* Group-level cultural evolution could be studied with an eye towards policy and intervention. However, nations and corporations probably evolve very differently. Nations are obligate and exclusive territorial groups while companies vary in their territorial claims. So, cultural group selection on countries should at least favour the maintenance of critical environmental resources within those territories. Indeed, countries do display a set of territorial resource traits including strategic energy (oil) reserves, agricultural supports and subsidies, and various environmental quality regulations. Companies, by contrast, are non-territorial groups. So cultural selection on companies should favour resource and energy acquisition regardless of external or deferred impacts. These differences may addressable through policy;
(v) *can global environmental governance emerge without an ETII?* The degree of global cooperation required for sustainable management of the global biosphere may be high (e.g. international economic and military treaties on environmental regulation), but it must be less than that required for a full ETII (e.g. group-level reproductive specialization). This suggests that an evolutionary transition is not necessary to solve near term global environmental challenges. Indeed, global environmental traits and laws have already emerged [92]. These include the Montreal Protocol, the United Nations Framework Convention on Climate Change, and the Paris Agreement. Detailed study of the conditions for the evolution of global self-governance is needed. Comparative case studies and theoretical modelling linking cultural evolution, economics and political science is needed; and
(vi) *can a human ETII be completed on a single planet, even in theory?* The population structure problem should be formally tested. New models could build on multi-level selection models with environmental resource constraints [24,131], ETI models with environmental patchiness [102,116] and cultural adaptation models with population size constraints [21,132]. These can enable new questions to be tested. For example, how could the forces and factors of cultural evolution be intentionally structured to improve the chances of the emergence of global cultural sustainability traits?

Finally, if our interpretation of the ETII hypothesis is valid, the problem of the Anthropocene is not just that humanity needs to solve collective environmental challenges at an unprecedented scale. It is that the central patterns of human evolution may prevent us from doing so. In this light, we propose a new definition of the Anthropocene as a period in human evolution:

the Anthropocene: *the period in which global environmental factors determine human evolutionary outcomes*.

The definition has five key features. First, it is not a geological epoch defined by stratigraphic features, but a novel period in human evolution defined by eco-evolutionary conditions. Second, this period is defined by the conditions in which individual human groups are sufficiently powerful to influence the global environment and thereby all other human groups. Third, this period entails a conflict between the scale of a society that could express the global cultural traits necessary to sustainably manage the global environment (i.e. global-scale society) and the human population structure necessary to evolve those traits (i.e. many such societies). Fourth, under the global environmental constraints of this period, the signature processes of group-level cultural evolution described by the ETII hypothesis may reduce the scope for the evolution of global environmental management traits. Fifth, the global constraint on human evolution endangers the completion of a human evolutionary transition and threatens the long-term persistence of our species.

## 9. Implications for policy and intervention in the Anthropocene

Our investigation leads us to suspect that the typical description of the challenges facing humanity in the Anthropocene is understated. When the patterns and processes of long-term human evolution in the environment are also considered, there is no clear and safe path through the Anthropocene. Nonetheless, our framework provides useful policy guidance to avoid near-term environmental disaster in a few ways.

First, the Anthropocene should be understood in terms of human evolution, and the ETII provides inspiration for new policy approaches and methods. We do not propose building policy solely from a new and untested theory. However, our mechanistic framework is an improvement over calls for a ‘crisis discipline’ of global collective behaviour [133] and GCR research [5,111], which have lacked mechanistic theory. Other theories of human evolution should be similarly explored. By integrating empirically validated and characteristic patterns of long-term human evolution with the collective behavioural requirements for global environmental sustainability, we can refine our estimation of the likelihood of catastrophic outcomes and develop useful guidance for policy exploration and inclusive sustainable solutions.

We suggest a simple and pragmatic approach: focus on solving the most pressing global environmental challenge of the moment. We do not need to solve the population structure problem of the ETII, at least not immediately. Similarly, we need not solve all the interconnected global environmental problems of the Anthropocene at once, although a sustainable global society must have that capacity. Right now, we need to solve the collective challenge of climate change. Then, we should turn to the next most pressing collective challenge and can keep solving collective global challenges for as long as we can.

Our study puts the role of cooperation and competition in human affairs in a different light than traditional economics and policy discussions. While growing global cooperation among societies may be the primary goal, cultural evolution via group competition is the evolutionary force that drives the most relevant adaptive change in human systems. This suggests, paradoxically, we must use competition among groups to build cooperation between groups. However, this may not be as far-fetched as it sounds. Perhaps we can use these two forces in careful concert to grow our collective capacity for global resource stewardship. For example, today's societies benefit from managed group-level cultural evolution in the form of peaceful competition through social systems such as markets and research grant competitions. Both generate socially valuable outputs. So, we could build intentional, peaceful and ethical systems of competitive cultural evolution to generate solutions for advancing global environmental cooperation.

For example, our study provides inspiration for solving climate change. We need to alter the direction of cultural selection on fossil fuel use among nations (via treaties) and companies (via market regulation). The means to accomplish this managed evolution are often equivalent to traditional policy approaches (e.g. carbon taxes and carbon tariffs [134]), climate clubs, investment in alternatives and bans on fossil fuel extraction and use). Evolutionary analysis simply provides an integrated theory and set of metrics. It also reminds us that these simple solutions may be the only real alternative to a spiraling pathway of increasingly direct conflict between groups.

## 10. Conclusion

In conclusion, connecting the Anthropocene and the ETII hypothesis has proved fruitful in both directions. The ETII helps to explain the human domination of the biosphere, from its evolutionary roots, to its current dynamics, to the shape of alternative paths we may chose. Meanwhile, the Anthropocene forces us consider if the ETII is likely to complete.

The ETII hypothesis proposes that human evolution has been dominated by feedbacks which accelerate group-level cultural adaptation and the intensity of group-level environmental control and impacts. This evolutionary ratchet has created the powerful niche-constructing groups that dominate human activity today, and the global-scale impacts they have generated. Human cultural evolution generally, and the ETII specifically, is the cause of the Anthropocene. This suggests that the sustainability and survival challenges of the Anthropocene are understated. The Anthropocene puts the processes that have steered human evolution for possibly millions of years in conflict with the evolutionary requirements for the global cultural traits we need.

Ours is a bleak reading of the possibilities of the future of environmental management and human evolution on Earth. However, it is useful because it is bleak. Worst-case scenarios are an indispensable planning tool (e.g. [5]). So, it may be on the intentional processes in cultural evolution, including innovation, foresight, planning and collective action, must be where we make our stand [135,136], by building global governance for the Anthropocene [94] even though it is against the interests of existing groups. It is our hope that this perspective can contribute to that collective effort, expanding the considerations of society today to help better select long-term paths in future.

We have suggested that humanity might be poorly adapted to survive a new evolutionary relationship to the biosphere. Even if this proposition is only slightly likely, or partially true, it deserves sharp attention. We hope that our raising the issue strikes new alarms and helps to motivate greater efforts at collective action.

## Data Availability

This article has no additional data.

## References

[1] Steffen W, Crutzen PJ, McNeill JR. 2007 The Anthropocene: are humans now overwhelming the great forces of nature. J. Hum. Environ. **36**, 614-621. (10.1579/0044-7447(2007)36[614:TAAHNO]2.0.CO;2) 18240674

[2] Waters CN et al. 2016 The Anthropocene is functionally and stratigraphically distinct from the Holocene. Science **351**, aad2622. (10.1126/science.aad2622) 26744408

[3] Ellis EC. 2015 Ecology in an anthropogenic biosphere. Ecol. Monogr. **85**, 287-331. (10.1890/14-2274.1)

[4] Wood ZT, Palkovacs EP, Olsen BJ, Kinnison MT. 2021 The importance of eco-evolutionary potential in the Anthropocene. BioScience **71**, 805-819. (10.1093/biosci/biab010)

[5] Kemp L et al. 2022 Climate endgame: exploring catastrophic climate change scenarios. Proc. Natl Acad. Sci. USA **119**, e2108146119. (10.1073/pnas.2108146119) 35914185 PMC9407216

[6] Ceballos G, Ehrlich PR, Dirzo R. 2017 Biological annihilation via the ongoing sixth mass extinction signaled by vertebrate population losses and declines. Proc. Natl Acad. Sci. USA **114**, E6089-E6096. (10.1073/pnas.1704949114) 28696295 PMC5544311

[7] Barnosky AD et al. 2011 Has the Earth's sixth mass extinction already arrived? Nature **471**, Article 7336. (10.1038/nature09678) 21368823

[8] Chapin FS et al. 2022 Earth stewardship: shaping a sustainable future through interacting policy and norm shifts. Ambio **51**, 1907-1920. (10.1007/s13280-022-01721-3) 35380347 PMC8982314

[9] Levin SA et al. 2022 Governance in the face of extreme events: lessons from evolutionary processes for structuring interventions, and the need to go beyond. Ecosystems **25**, 697-711. (10.1007/s10021-021-00680-2) 34512142 PMC8422834

[10] Beddoe R et al. 2009 Overcoming systemic roadblocks to sustainability: the evolutionary redesign of worldviews, institutions, and technologies. Proc. Natl Acad. Sci. USA **106**, 2483-2489. (10.1073/pnas.0812570106) 19240221 PMC2650289

[11] Ehrlich PR, Ornstein RE. 2010 Humanity on a tightrope: thoughts on empathy, family, and big changes for a viable future. New York, NY: Rowman & Littlefield Publishers.

[12] Van Den Bergh JC. 2018 Human evolution beyond biology and culture: evolutionary social, environmental and policy sciences. Cambridge, UK: Cambridge University Press.

[13] Waring T, Kline M, Brooks J, Goff S, Gowdy J, Janssen M, Smaldino P, Jacquet J. 2015 A multilevel evolutionary framework for sustainability analysis. Ecol. Soc. **20**, 34. (10.5751/ES-07634-200234)

[14] Richerson PJ, Boyd R. 2005 Not by genes alone: how culture transformed human evolution. Chicago, IL: University of Chicago Press.

[15] Schill C et al. 2019 A more dynamic understanding of human behaviour for the Anthropocene. Nat. Sustain. **2**, 12. (10.1038/s41893-019-0419-7)

[16] Ehrlich PR, Ehrlich AH. 2022 Returning to ‘Normal’? Evolutionary roots of the human prospect. BioScience **72**, 778-788. (10.1093/biosci/biac044) 35923190 PMC9343229

[17] Wilson RS, Herziger A, Hamilton M, Brooks JS. 2020 From incremental to transformative adaptation in individual responses to climate-exacerbated hazards. Nat. Clim. Change **10**, 200-208. (10.1038/s41558-020-0691-6)

[18] Adger WN, Barnett J, Brown K, Marshall N, O'Brien K. 2013 Cultural dimensions of climate change impacts and adaptation. Nat. Clim. Change **3**, 2. (10.1038/nclimate1666)

[19] Kaaronen RO, Borgerhoff Mulder M, Waring TM. 2022 *Applying cultural evolution to address climate and environmental challenges*. OSF Preprints. (10.31219/osf.io/u7hvj)

[20] Richerson P et al. 2016 Cultural group selection plays an essential role in explaining human cooperation: a sketch of the evidence. Behav. Brain Sci. **39**, e30 (19 pages). (10.1017/S0140525X1400106X) 25347943

[21] Henrich J. 2004 Demography and cultural evolution: how adaptive cultural processes can produce maladaptive losses—the Tasmanian Case. Am. Antiquity **69**, 197-214. (10.2307/4128416)

[22] Safarzyńska K, Frenken K, Van Den Bergh JC. 2012 Evolutionary theorizing and modeling of sustainability transitions. Evol. Hum. Behav. **41**, 1011-1024. (10.1016/j.respol.2011.10.014)

[23] Ellis EC, Magliocca NR, Stevens CJ, Fuller DQ. 2018 Evolving the Anthropocene: linking multi-level selection with long-term social–ecological change. Sustainability Sci. **13**, 119-128. (10.1007/s11625-017-0513-6) PMC6086254 30147774

[24] Waring TM, Goff SH, Smaldino PE. 2017 The coevolution of economic institutions and sustainable consumption via cultural group selection. Ecol. Econ. **131**, 524-532. (10.1016/j.ecolecon.2016.09.022)

[25] Safarzyńska K. 2013 Evolutionary-economic policies for sustainable consumption. Ecol. Econ. **90**, 187-195. (10.1016/j.ecolecon.2013.03.020)

[26] Waring T, Acheson J. 2018 Evidence of cultural group selection in territorial lobstering in Maine. Sustainability Sci. **13**, 21-34. (10.1007/s11625-017-0501-x) PMC6086256 30147768

[27] Andrews J, Borgerhoff Mulder M. 2018 Cultural group selection and the design of REDD+: insights from Pemba. Sustainability Sci. **13**, 93-107. (10.1007/s11625-017-0489-2) PMC6086255 30147773

[28] Pisor AC, Surbeck M. 2019 The evolution of intergroup tolerance in nonhuman primates and humans. Evol. Anthropol. **28**, 210-223. (10.1002/evan.21793) 31386248

[29] Pisor AC, Borgerhoff Mulder M, Smith KM. 2023 Long-distance social relationships can both undercut and promote natural resource management. Phil. Trans. R. Soc. B **378**, 20220269. (10.1098/rstb.2022.0269) PMC10645093 37952627

[30] Waring TM, Wood ZT. 2021 Long-term gene–culture coevolution and the human evolutionary transition. Proc. R. Soc. B **288**, 20210538. (10.1098/rspb.2021.0538) PMC8170228 34074122

[31] Currie TE, Borgerhoff Mulder M, Fogarty L, Schlüter M, Haider LJ, Tavoni A, Jansen R, Folke C, Waring TM. 2023 Integrating evolutionary theory and social-ecological systems research to address the sustainability challenges of the Anthropocene. Phil. Trans. R. Soc. B **378**, 20220262. (doi:10.1098.2022.0262) 10.1098/rstb.2022.0262 PMC10645068 37952618

[32] Maynard Smith J, Szathmáry E. 1995 The major transitions in evolution. Oxford, UK: Oxford University Press.

[33] Maynard Smith J, Szathmáry E. 2000 The origins of life: from the birth of life to the origin of language. Oxford, UK: Oxford University Press.

[34] Stearns SC. 2007 Are we stalled part way through a major evolutionary transition from individual to group? Evolution **61**, 2275-2280. (10.1111/j.1558-5646.2007.00202.x) 17910743

[35] Powers ST, van Schaik CP, Lehmann L. 2016 How institutions shaped the last major evolutionary transition to large-scale human societies. Phil. Trans. R. Soc. B **371**, 20150098. (10.1098/rstb.2015.0098) 26729937 PMC4760198

[36] Andersson C, Törnberg P. 2018 Toward a macroevolutionary theory of human evolution: the social protocell. Biol. Theory **14**, 86-102. (10.1007/s13752-018-0313-y)

[37] Bickerton D. 2009 Adam's tongue: how humans made language, how language made humans. New York, NY: Macmillan.

[38] Andersson C, Czárán T. 2023 The transition from animal to human culture—simulating the social protocell hypothesis. Phil. Trans. R. Soc. B **378**, 20210416. (10.1098/rstb.2021.0416) 36688383 PMC9869448

[39] Rainey PB. 2023 Major evolutionary transitions in individuality between humans and AI. Phil. Trans. R. Soc. B **378**, 20210408. (10.1098/rstb.2021.0408) 36688400 PMC9869444

[40] Jablonka E. 1994 Inheritance Systems and the Evolution of New Levels of Individuality. J. Theoretical Biol. **170**, 301-309. (10.1006/jtbi.1994.1191) 7996858

[41] Perreault C. 2012 The pace of cultural evolution. PLoS ONE **7**, e45150. (10.1371/journal.pone.0045150) 23024804 PMC3443207

[42] Mathew S, Perreault C. 2015 Behavioural variation in 172 small-scale societies indicates that social learning is the main mode of human adaptation. Proc. R. Soc. B **282**, 20150061. (10.1098/rspb.2015.0061) PMC4590464 26085589

[43] Michod RE. 2005 On the transfer of fitness from the cell to the multicellularorganism. Biol. Phil. **20**, 967-987. (10.1007/s10539-005-9018-2)

[44] Davison D, Andersson C, Michod R, Kuhn S. 2021 Did human culture emerge in a cultural evolutionary transition in individuality? Biol. Theory **16**, 213-236. (10.1007/s13752-021-00382-x)

[45] Okasha S. 2006 Evolution and the levels of selection. Oxford, UK: Oxford University Press.

[46] Crespi BJ. 2004 Vicious circles: positive feedback in major evolutionary and ecological transitions. Trends Ecol. Evol. **19**, 627-633. (10.1016/j.tree.2004.10.001) 16701324

[47] Francois P, Fujiwara T, van Ypersele T. 2018 The origins of human prosociality: cultural group selection in the workplace and the laboratory. Sci. Adv. **4**, eaat2201. (10.1126/sciadv.aat2201) 30255142 PMC6154982

[48] Handley C, Mathew S. 2020 Human large-scale cooperation as a product of competition between cultural groups. Nat. Commun. **11**, 702. (10.1038/s41467-020-14416-8) 32019930 PMC7000669

[49] Carmel Y. 2023 Human societal development: is it an evolutionary transition in individuality? Phil. Trans. R. Soc. B **378**, 20210409. (10.1098/rstb.2021.0409) 36688399 PMC9869447

[50] Tennie C, Call J, Tomasello M. 2009 Ratcheting up the ratchet: on the evolution of cumulative culture. Phil. Trans. R. Soc. B **364**, 2405-2415. (10.1098/rstb.2009.0052) 19620111 PMC2865079

[51] Tomasello M, Kruger AC, Ratner HH. 1993 Cultural learning. Behav. Brain Sci. **16**, 495-511. (10.1017/S0140525X0003123X)

[52] van Gestel J, Tarnita CE. 2017 On the origin of biological construction, with a focus on multicellularity. Proc. Natl Acad. Sci. USA **114**, 11 018-11 026. (10.1073/pnas.1704631114) 28973893 PMC5651743

[53] Hawkes K, O'Connell JF, Blurton Jones NG, Oftedal OT, Blumenschine RJ, Widdowson EM, Whiten A, Bone Q. 1991 Hunting income patterns among the Hadza: big game, common goods, foraging goals and the evolution of the human diet. Phil. Trans. R. Soc. Lond. B **334**, 243-251. (10.1098/rstb.1991.0113) 1685582

[54] Næss MW. 2020 Cultural group selection and the evolution of reindeer herding in Norway. Hum. Ecol. **48**, 279-291. (10.1007/s10745-020-00158-0)

[55] Altman A, Mesoudi A. 2019 Understanding agriculture within the frameworks of cumulative cultural evolution, gene-culture co-evolution, and cultural niche construction. Hum. Ecol. **47**, 483-497. (10.1007/s10745-019-00090-y)

[56] Chase JM, Leibold MA. 2003 Ecological niches: linking classical and contemporary approaches. Chicago, IL: University of Chicago Press. See https://press.uchicago.edu/ucp/books/book/chicago/E/bo3638660.html.

[57] Odling-Smee J, Erwin DH, Palkovacs EP, Feldman MW, Laland KN. 2013 Niche construction theory: a practical guide for ecologists. Q. Rev. Biol. **88**, 3-28. (10.1086/669266) 23653966

[58] Laland KN, Odling-Smee J, Feldman MW. 2000 Niche construction, biological evolution, and cultural change. Behav. Brain Sci. **23**, 131-146. (10.1017/S0140525X00002417) 11303338

[59] Laland KN, Odling-Smee J, Feldman MW. 2001 Cultural niche construction and human evolution. J. Evol. Biol. **14**, 22-33. (10.1046/j.1420-9101.2001.00262.x) 29280584

[60] Laland KN, O'Brien MJ. 2011 Cultural niche construction: an introduction. Biol. Theory **6**, 191-202. (10.1007/s13752-012-0026-6)

[61] Morris I. 2010 Why the west rules–for now: the patterns of history, and what they reveal about the future. New York: NY: Farrar, Straus and Giroux.

[62] Klein Goldewijk K, Beusen A, Doelman J, & SE. 2017 Anthropogenic land use estimates for the Holocene – HYDE 3.2. Earth Syst. Sci. Data **9**, 927-953. (10.5194/essd-9-927-2017)

[63] Mongle CS, Strait DS, Grine FE. 2023 An updated analysis of hominin phylogeny with an emphasis on re-evaluating the phylogenetic relationships of *Australopithecus sediba*. J. Hum. Evol. **175**, 103311. (10.1016/j.jhevol.2022.103311) 36706599

[64] Plummer TW et al. 2023 Expanded geographic distribution and dietary strategies of the earliest Oldowan hominins and Paranthropus. Science **379**, 561-566. (10.1126/science.abo7452) 36758076

[65] Hershkovitz I et al. 2018 The earliest modern humans outside Africa. Science **359**, 456-459. (10.1126/science.aap8369) 29371468

[66] Lobo J, Whitelaw T, Bettencourt LMA, Wiessner P, Smith ME, Ortman S. 2022 Scaling of hunter-gatherer camp size and human sociality. Curr. Anthropol. **63**, 68-94. (10.1086/719234)

[67] Ferraro JV et al. 2013 Earliest archaeological evidence of persistent hominin carnivory. PLoS ONE **8**, e62174. (10.1371/journal.pone.0062174) 23637995 PMC3636145

[68] Pobiner BL. 2020 The zooarchaeology and paleoecology of early hominin scavenging. Evol. Anthropol. **29**, 68-82. (10.1002/evan.21824) 32108400

[69] Thompson JC, Carvalho S, Marean CW, Alemseged Z. 2019 Origins of the human predatory pattern: the transition to large-animal exploitation by early hominins. Curr. Anthropol. **60**, 1-23. (10.1086/701477)

[70] Stiner MC, Barkai R, Gopher A. 2009 Cooperative hunting and meat sharing 400–200 kya at Qesem Cave, Israel. Proc. Natl Acad. Sci. USA **106**, 13 207-13 212. (10.1073/pnas.0900564106) PMC2726383 19666542

[71] Goren-Inbar N, Alperson N, Kislev ME, Simchoni O, Melamed Y, Ben-Nun A, Werker E. 2004 Evidence of hominin control of fire at Gesher Benot Yàaqov, Israel. Science **304**, 725-727. (10.1126/science.1095443) 15118160

[72] Zeder MA. 2017 Domestication as a model system for the extended evolutionary synthesis. Interface Focus **7**, 20160133. (10.1098/rsfs.2016.0133) 28839915 PMC5566803

[73] Vitousek PM, Mooney HA, Lubchenco J, Melillo JM. 1997 Human domination of Earth's ecosystems. Science **277**, 494-499. (10.1126/science.277.5325.494)

[74] Palumbi SR. 2001 Humans as the World's greatest evolutionary force. Science **293**, 1786-1790. (10.1126/science.293.5536.1786) 11546863

[75] Bickerton D, Szathmáry E. 2011 Confrontational scavenging as a possible source for language and cooperation. BMC Evol. Biol. **11**, 261. (10.1186/1471-2148-11-261) 21933413 PMC3188516

[76] Szilágyi A, Kovács VP, Czárán T, Szathmáry E. 2023 Evolutionary ecology of language origins through confrontational scavenging. Phil. Trans. R. Soc. B **378**, 20210411. (10.1098/rstb.2021.0411) 36688391 PMC9869442

[77] Andermann T, Faurby S, Turvey ST, Antonelli A, Silvestro D. 2020 The past and future human impact on mammalian diversity. Sci. Adv. **6**, eabb2313. (10.1126/sciadv.abb2313) 32917612 PMC7473673

[78] Barnosky AD. 2008 Megafauna biomass tradeoff as a driver of Quaternary and future extinctions. Proc. Natl Acad. Sci. USA **105**(Suppl. 1), 11 543-11 548. (10.1073/pnas.0801918105) 18695222 PMC2556404

[79] Stiner MC. 2002 Carnivory, coevolution, and the geographic spread of the genus *Homo*. J. Archaeol. Res. **10**, 1-63. (10.1023/A:1014588307174)

[80] Perri AR, Feuerborn TR, Frantz LAF, Larson G, Malhi RS, Meltzer DJ, Witt KE. 2021 Dog domestication and the dual dispersal of people and dogs into the Americas. Proc. Natl Acad. Sci. USA **118**, e2010083118. (10.1073/pnas.2010083118) 33495362 PMC8017920

[81] Clutton-Brock J. 1999 A review of the natural history of domesticated mammals. London, UK: Natural History Museum.

[82] Zeder MA. 2011 The origins of agriculture in the near east. Curr. Anthropol. **52**, S221-S235. (10.1086/659307)

[83] Zohary D, Hopf M. 2000 Domestication of plants in the Old world: the origin and spread of cultivated plants in west Asia, Europe and the Nile valley, 3rd edn. Oxford, UK: Oxford University Press. See https://www.cabdirect.org/cabdirect/abstract/20013014838

[84] Stephens L et al. 2019 Archaeological assessment reveals Earth's early transformation through land use. Science **365**, 897-902. (10.1126/science.aax1192) 31467217

[85] Rockström J et al. 2009 Planetary boundaries: exploring the safe operating space for humanity. Ecol. Soc. **14**, 1-33. (10.5751/ES-03180-140232)

[86] Choi JK, Bowles S. 2007 The coevolution of parochial altruism and war. Science **318**, 636-640. (10.1126/science.1144237) 17962562

[87] Turchin P. 2016 Ultrasociety: how 10,000 years of war made humans the greatest cooperators on earth. Chaplin, CT: Beresta Books.

[88] Makan A. 2007 Island nations plan for rising seas, mass migration. *Reuters*. See https://www.reuters.com/article/idUSSP277709

[89] UNESCAP. 2022 Pacific climate change and migration project*.* (Project report). See https://www.unescap.org/subregional-office/pacific/pacific-climate-change-and-migration-project.

[90] Thomas AR, Fulkerson GM. 2021 City and country: the historical evolution of urban-rural systems. Lanham, MD: Rowman & Littlefield.

[91] Henderson DA. 2012 A history of eradication—successes, failures, and controversies. The Lancet **379**, 884-885. (10.1016/S0140-6736(12)60381-X)

[92] Yang T, Percival RV. 2009 The emergence of global environmental law. Ecol. Law Q. **36**, 615-664.

[93] Kotzé L. 2019 A global environmental constitution for the Anthropocene? Trans. Environ. Law **8**, 11-33. (10.1017/S2047102518000274)

[94] Woolley O, Harrington C. 2022 Law and governance in the Anthropocene. Glob. Policy **13**, 5-10. (10.1111/1758-5899.13168)

[95] Corning PA. 2022 Politics in evolution: the first 5 million years, and the next 100. In Biopolitics at 50 years, vol. 13 (eds T Wohlers, A Fletcher), pp. 27-45. Bingley, UK: Emerald Publishing Limited.

[96] Lovelock JE, Margulis L. 1974 Atmospheric homeostasis by and for the biosphere: the Gaia hypothesis. Tellus **26**, 2-10. (10.1111/j.2153-3490.1974.tb01946.x)

[97] Tyrrell T. 2013 On Gaia: a critical investigation of the relationship between life and earth. Princeton, NJ: Princeton University Press.

[98] Lenton TM. 2004 Clarifying Gaia: regulation with or without natural selection. In Scientists debate Gaia: the next century (eds SH Schneider, JR Miller, E Crist, PJ Boston). Cambridge, MA: The MIT Press.

[99] Doolittle WE. 2014 Natural selection through survival alone, and the possibility of Gaia. Biol. Phil. **29**, 415-423. (10.1007/s10539-013-9384-0)

[100] Doolittle WF. 2019 Making evolutionary sense of Gaia. Trends Ecol. Evol. **34**, 889-894. (10.1016/j.tree.2019.05.001) 31155421

[101] Bernhardt JR, Kratina P, Pereira AL, Tamminen M, Thomas MK, Narwani A. 2020 The evolution of competitive ability for essential resources. Phil. Trans. R. Soc. B **375**, 20190247. (10.1098/rstb.2019.0247) 32200736 PMC7133530

[102] Black AJ, Bourrat P, Rainey PB. 2020 Ecological scaffolding and the evolution of individuality. Nat. Ecol. Evol. **4**, Article 3. (10.1038/s41559-019-1086-9) 32042121

[103] Ostrom E. 1990 Governing the commons: the evolution of institutions for collective action. Cambridge, UK: Cambridge University Press.

[104] Haasnoot M, Kwakkel JH, Walker WE, ter Maat J. 2013 Dynamic adaptive policy pathways: a method for crafting robust decisions for a deeply uncertain world. Glob. Environ. Change **23**, 485-498. (10.1016/j.gloenvcha.2012.12.006)

[105] Diamond J. 2011 Collapse: how societies choose to fail or succeed: revised edition. Harmondsworth, UK: Penguin.

[106] Tainter JA. 1996 Complexity, problem solving, and sustainable societies. In Getting Down to Earth: Practical Applications of Ecological Economics (eds R Costanza, O Segura, J Martinez-Alier), pp. 61-76. Washington, DC: Island Press.

[107] Tainter J. 1988 The collapse of complex societies. Cambridge, UK: Cambridge University Press.

[108] Zefferman MR, Mathew S. 2015 An evolutionary theory of large-scale human warfare: group-structured cultural selection. Evol. Anthropol. **24**, 50-61. (10.1002/evan.21439) 25914359

[109] Turchin P et al. 2022 Disentangling the evolutionary drivers of social complexity: a comprehensive test of hypotheses. Sci. Adv. **8**, eabn3517. (10.1126/sciadv.abn3517) 35749491 PMC9232109

[110] Vesco P, Dasgupta S, De Cian E, Carraro C. 2020 Natural resources and conflict: a meta-analysis of the empirical literature. Ecol. Econ. **172**, 106633. (10.1016/j.ecolecon.2020.106633)

[111] Fisher L, Sandberg A. 2022 A safe governance space for humanity: necessary conditions for the governance of global catastrophic risks. Glob. Policy **13**, 792-807. (10.1111/1758-5899.13030) 37056960 PMC10084266

[112] Søgaard Jørgensen P et al. 2023 Evolutionary traps for humanity in the Anthropocene and the pursuit of global sustainability. Phil. Trans. R. Soc. B **378**, 20220261. (10.1098/rstb.2022.0261)

[113] Zhang RJ, Liu JH, Lee M, Lin M, Xie T. 2023 Continuities and discontinuities in the cultural evolution of global consciousness. Phil. Trans. R. Soc. B **378**, 20220263. (10.1098/rstb.2022.0263) PMC10645106 37952613

[114] Hardin G. 1960 The competitive exclusion principle. Science **131**, 1292-1297. (10.1126/science.131.3409.1292) 14399717

[115] Blegen N. 2017 The earliest long-distance obsidian transport: evidence from the ∼200 ka Middle Stone Age Sibilo School Road Site, Baringo, Kenya. J. Human Evol. **103**, 1-19. (10.1016/j.jhevol.2016.11.002) 28166905

[116] Nitschke MC, Black AJ, Bourrat P, Rainey PB. 2023 The effect of bottleneck size on evolution in nested Darwinian populations. J. Theoret. Biol. **561**, 111414. (10.1016/j.jtbi.2023.111414) 36639021

[117] Michod RE. 2007 Evolution of individuality during the transition from unicellular to multicellular life. Proc. Natl Acad. Sci. USA **104**(Suppl. 1), 8613-8618. (10.1073/pnas.0701489104) 17494748 PMC1876437

[118] Chouvenc T, Šobotník J, Engel MS, Bourguignon T. 2021 Termite evolution: mutualistic associations, key innovations, and the rise of Termitidae. Cell. Mol. Life Sci. **78**, 2749-2769. (10.1007/s00018-020-03728-z) 33388854 PMC11071720

[119] Waring T, Lange T, Chakraborty S. 2021 Institutional adaptation in the evolution of the ‘co-operative principles.’ J. Evol. Economics. (10.1007/s00191-021-00738-3)

[120] Turchin P et al. 2015 *Seshat: The Global History Databank*. See http://uhra.herts.ac.uk/handle/2299/16139.

[121] Snyder BF. 2020 The genetic and cultural evolution of unsustainability. Sustainability Science **15**, 1087-1099. (10.1007/s11625-020-00803-z) 32292525 PMC7133775

[122] Kemp L. 2019 Are we on the road to civilisation collapse? *BBC Future*. See https://www.bbc.com/future/article/20190218-are-we-on-the-road-to-civilisation-collapse.

[123] Taagepera R. 1979 Size and duration of empires: growth-decline curves, 600 B.C. to 600 A.D. Soc. Sci. Hist. **3**, 115-138. (10.2307/1170959)

[124] Taagepera R. 1978 Size and duration of empires growth-decline curves, 3000 to 600 b.c. Soc. Sci. Res. **7**, 180-196. (10.1016/0049-089X(78)90010-8)

[125] Dun & Bradstreet. 2017 *The Dun & Bradstreet D-U-N-S® Number The Universal Standard for Business Identification*. 2.

[126] Hillenbrand P, Kiewell D, Miller-Cheevers R, Ostojic I, Springer G. 2019 *Traditional company, new businesses: the pairing that can ensure an incumbent's survival*, p. 11. New York, NY: McKinsey & Company.

[127] Crippa M et al. 2022 CO2 emissions of all world countries, EUR 31182 EN. Luxembourg: Publications Office of the European Union. (10.2760/730164)

[128] UNDP. 2022 Human Development Report 2021–22. New York, NY: United Nations. See https://hdr.undp.org/content/human-development-report-2021-22.

[129] Barbato A, Kenny E, Weiner PM, Pringle T, Vollmer ME, Fagerlin D, Bailey J. 2021 Fortune 500 Emissions Report FY2019 (Fortune 500 Companies Greenhouse Gas Emissions). Recapture. See https://www.recapturecarbon.com/fortune-500-emissions-report-2019.

[130] Largest US Corporations: Fortune Five Hundred. (15 June 2015). *Fortune*, F1–F22.

[131] Safarzynska K, Smaldino PE. 2023 Reducing global inequality increases cooperation: a simple model of group selection with a global externality. Phil. Trans. R. Soc. B **378**, 20220267. (10.1098/rstb.2022.0267) PMC10645090 37952620

[132] Lake MW, Crema ER. 2012 The cultural evolution of adaptive-trait diversity when resources are uncertain and finite. Adv. Complex Syst. **15**, 1150013. (10.1142/S0219525911003323)

[133] Bak-Coleman JB et al. 2021 Stewardship of global collective behavior. Proc. Natl Acad. Sci. USA **118**, e2025764118. (10.1073/pnas.2025764118) 34155097 PMC8271675

[134] Abnett K. 2022 EU strikes deal on world-first carbon border tariff. *Reuters*. See https://www.reuters.com/markets/carbon/eu-strikes-deal-world-first-carbon-border-tariff-2022-12-13/.

[135] Richerson PJ, Boyd R, Efferson C. 2023 Agentic processes in cultural evolution: relevance to Anthropocene sustainability. Phil. Trans. R. Soc. B **378**, 20220252. (10.1098/rstb.2022.0252) PMC10645076 37952614

[136] Lenton TM, Latour B. 2018 Gaia 2.0. Science **361**, 1066-1068. (10.1126/science.aau0427) 30213897

